# Improving Mechanical Properties of Fe-Mn-Co-Cr High-Entropy Alloy via Annealing after Cold Rolling

**DOI:** 10.3390/ma17030676

**Published:** 2024-01-30

**Authors:** Yukun Lv, Pingtao Song, Yuzhe Wang, Xuerou Zhao, Wei Gao, Jie Zhang, Yutian Lei, Jian Chen

**Affiliations:** 1 School of Materials Science and Chemical Engineering, Xi’an Technological University, Xi’an 710021, China; pingtao123@163.com (P.S.); yuzhewan111@163.com (Y.W.); gaowei@163.com (W.G.); jie@yeah.net (J.Z.); lyt222@163.com (Y.L.); 2 School of Railway Equipment Manufacturing, Shaanxi Railway Institute, Weinan 714099, China; 18609237603@163.com

**Keywords:** microstructure, HEA, annealing, deformation twin

## Abstract

The as-cast (Fe50Mn30Co10Cr10)97C2Mo1 HEA (high entropy alloy) was prepared and cold-rolled at 70%. Subsequently, annealing heat treatment at different temperatures (900 °C, 950 °C, 1000 °C) was carried out. The microstructure evolution and mechanical properties of the HEA were systematically investigated. The results showed that the HEA annealed at 900 °C and 950 °C exhibited uneven grain size and rich σ precipitation phase at grain boundaries. The grains began to grow and complete recrystallization, and no σ phases were observed in HEA annealed at 1000 °C, which resulted in a higher tensile strength of ~885 MPa and elongation of ~68% compared with other annealed HEAs. The higher volume fraction of annealing twins with 60°<111> orientation was produced in HEA annealed at 1000 °C, which enhanced the tensile strength and plasticity via the Twinning-induced plasticity (TWIP) mechanism.

## 1. Introduction

High-entropy alloys (HEAs) have garnered significant interest in recent years [1,2]. Defined by a single-phase solid solution alloy composed of five elements in either an equimolar ratio or non-equimolar ratio, HEAs exhibit distinct characteristics such as high entropy, severe lattice distortion, sluggish diffusion, and cocktail effects as compared to conventional alloys [3,4,5]. Due to their unique design concept different from traditional alloys, their synthetic mechanical properties are excellent [6,7,8], mainly exhibiting high hardness, high ductility, good thermal stability, wear resistance, and corrosion resistance.

However, conventional metallic structural materials (e.g., steel, Al alloys) often show a trade-off relationship between strength and ductility [9,10,11,12]. Grain refinement, micro-strip-induced plasticity (MBIP), twin-induced plasticity (TWIP), and transformation-induced plasticity (TRIP) are important deformation mechanisms used to strengthen and toughen materials to conquer the trade-offs. Therefore, achieving a good combination of strength and ductility is a goal for many researchers in the material community. In a new study [13], it was shown that the interstitial carbon atoms were dissolved into the matrix of Fe50Mn30Co10Cr10 dual-phase HEA, and the strengthening of the solution occurred during plastic deformation. The TWIP (twinning-induced plasticity) effect was simultaneously excited. For example, adding 1.1 at.% carbon to Fe40.4Ni11.3Mn34.8Al7.5Cr6 HEA simultaneously improved the yield strength and ductility [14]. Carbon is often considered to be a stabilizer for the FCC phase, and it rises stacking fault energy (SFE) [15]. On the contrary, it has been reported that the addition of Mo decreases the SFE of Co-Cr-Fe-Ni HEA [16]. The introduction of carbon atoms into HEAs can reduce the SFE effectively and change the mode of dislocation slipping during plastic deformation. Additionally, carbon atoms increase the work hardening rate and delay the necking phenomenon. On the contrary, Mo can decrease the SFE when added to Fe-Mn TWIP alloys [17]. Accordingly, adding interstitial strengthening elements (C\Mo) affect the SFE and deformation mechanism. 

Moreover, thermal–mechanical processing is an effective method to eliminate some as-cast defects, refine the structure, and promote the formation of precipitates, thus optimizing the microstructure and improving mechanical properties [18,19,20]. For instance, annealing for the rolled sample produces complete, recrystallized, ultrafine grains and annealing twins, thus improving the strength and ductility [21]. Li [22] studied the effect of annealing on 40% cold-rolled Al0.5CoFeCrNiSi0.25 dual-phase, high-entropy alloys (DHEAs) and found that annealing for 1 h at 1100 °C produced a good combination of tensile strength (~1267 MPa) and ductility (uniform elongation of ~34.4%). Accordingly, thermomechanical processing, such as rolling + annealing, is an effective way to tailor the microstructures and mechanical properties of HEAs [23,24]. In our previous study, a good strength–ductility balance of (Fe50Mn30Co10Cr10)97C2Mo1 HEA was designed and achieved [25]. However, the yield strength of this HEA was only 425 MPa, and thermomechanical methods at different temperatures were not used. In this paper, HEA with a composition of (Fe50Mn30Co10Cr10)97C2Mo1 (at.%) was selected by us, then the cold-rolled and annealed at different temperatures. Finally, the microstructures and mechanical properties after this thermomechanical processing were systematically investigated.

## 2. Materials and Methods

The (Fe50Mn30Co10Cr10)97C2Mo1 (at.%) (HEA) ingots were prepared through the arc-melting of pure elements (≥99.9 wt%) under a Ti-gettered, high-purity argon atmosphere in a water-cooled Cu crucible. The ingots were melted five times to achieve good homogeneity. The as-casted HEA was solution-treated at 1200 °C for 2 h and then water quenched to obtain a complete uniform microstructure. The homogenized ingots were subjected to the following processes: hot rolling with a reduction of 20% at 1100 °C; cold rolling with a reduction of ~70%; and then annealing at 900 °C, 950 °C, and 1000 °C for 5 min, respectively, hereafter designated as CR70, A900, A950, and A1000, respectively. Flat dog-bone-shaped specimens with a gauge length of 22 mm and a cross-section of 2.5 × 1.5 mm were cut using electrical discharge machining. Tensile tests were carried out on a UTM5105 electronic universal testing machine. A Lab XRD-6000 X-ray diffraction (XRD) (Bruker AXS, Karlsruhe, Germany) with Cu Ka radiation, operated at a scanning rate of 0.2°/min from 20° to 100° under 40 kV and 40 mA, was adopted to determine the crystal structure. The microstructure was characterized using a FEI Quanta-400F scanning electron microscope (SEM) (Thermo Fisher Scientific, Waltham, MA, USA) and a JEOL JEM-2010 transmission electron microscope (TEM) (JEOL Ltd., Tokyo, Japan). Electron backscattered diffraction (EBSD) analysis was performed at 20 kV with a working distance of 18 mm and a tilt angle of 70°. The analysis software (HKLCHANNEL 5 version 5.0.9.0) was utilized to interpret the EBSD data.

## 3. Results and Discussion

**Figure 1:**
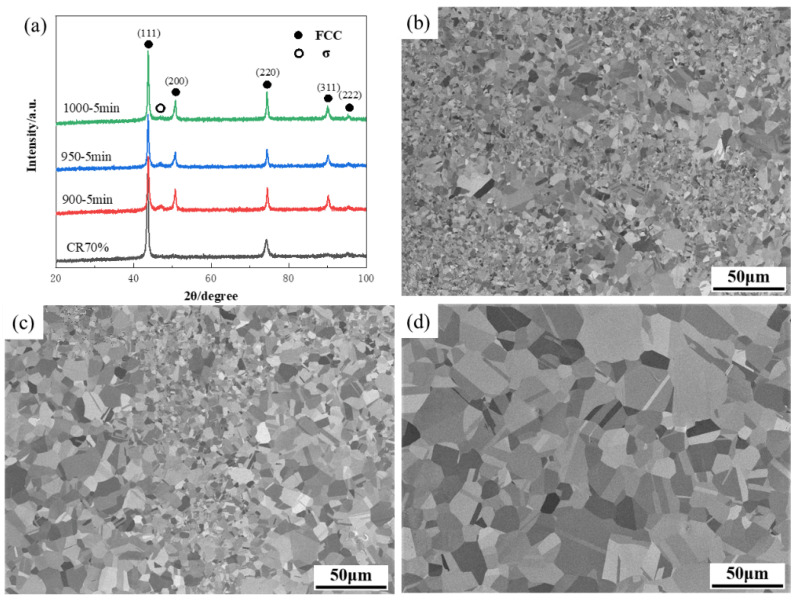
XRD and SEM images of the HEAs annealed at different temperatures. (**a**) XRD, (**b**) A900, (**c**) A950, (**d**) A1000.

The XRD patterns of the annealed HEA are shown in Figure 1a. The diffraction peaks corresponding to the FCC (face center cubic) phase appeared in CR70 and A1000 HEAs, indicating that no precipitation or intermetallic compounds were produced during the annealing process. However, the corresponding diffraction peaks of the σ phase, rich in Cr, were generated in the A900 and A950 HEAs, and then disappeared in the A1000 HEA. Figure 1b–d show SEM-BSE images annealing at 900 °C, 950 °C, and 1000 °C, respectively. It can be seen that, after annealing at 900 °C and 950 °C, the recrystallized grain size of the alloy was uneven, and different forms of annealing twins were formed in some large grains. At the same time, a large number of white elliptical particles precipitated at the grain boundaries, which were in the σ phase and rich in Cr. After being annealed at 1000 °C, the grains begin to grow and complete recrystallization, and no σ phases were observed at the grain boundaries. An interesting finding is that the more uniform equiaxed grains with small numbers of twins were formed in A1000 HEA, as shown in Figure 1d. Through the relationship between grain size, annealing temperature, and annealing time, the classical theory of grain growth kinetics can be used to calculate the activation energy in grain growth for (Fe50Mn30Co10Cr10) 97C2Mo1 HEA. The calculation formula is as follows [21,22]:

(1) $$d^{n}\;-\;d_{0}^{n}=kt$$

(2) $$k=k_{0}\;*\;e^{-\frac{Q}{RT}}$$

(3) $$\ln\left(\frac{d^{n}\;-\;d_{0}^{n}}{t}\right)=\ln k_{0}\;-\;\frac{Q}{RT}$$

In the above equation, *d*_0_ is the grain size before the holding time, t is the holding time, and d is the grain size after the holding time. K is a constant, R is a gas constant, T is the holding temperature, n is taken as 3, and Q is the activation energy required for grain growth. After calculation, the activation energy of the (Fe50Mn30Co10Cr10) 97C2Mo1 HEA was about 468 KJ/mol, higher than the previously reported CoCrFeMnNi HEA (325 KJ/mol), and three times that of austenitic 304 stainless steel (150 KJ/mol). The high activation energy of grain growth demonstrates the high diffusion hysteresis effect of HEA. Therefore, to achieve complete recrystallization of the alloy, a higher annealing temperature or sufficient annealing time was required.

**Figure 2:**
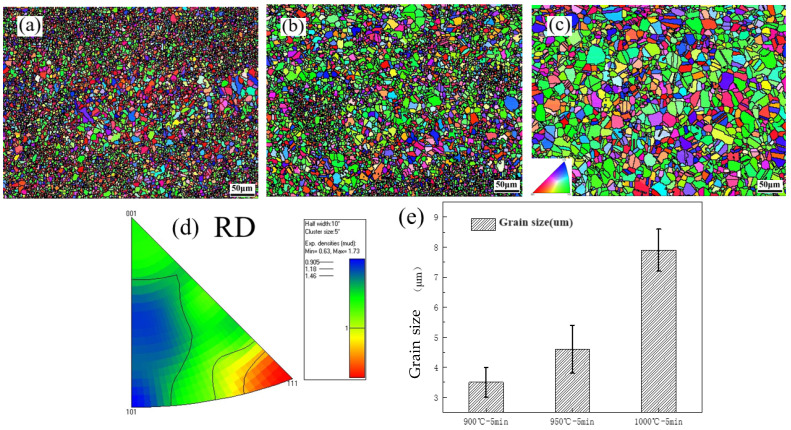
IPF diagram and grain size distribution diagram. (**a**) A900, (**b**) A950, (**c**) A1000. (**d**) Reverse pole diagram corresponding to A1000 HEA, (**e**) grain size distribution diagram.

Figure 2 shows the IPF diagram and grain size distribution diagram of HEAs. it can be seen that, in A900 HEA, there were more 3.5 μm small-sized grains with annealing twins. As the annealing temperature increased, the grain size increased to 4.6 μm in A950 HEA. Equiaxed grains were formed, and the grain size distribution increased to 7.9 μm after annealing at 1000 °C. At that moment, the annealed twins were uniformly distributed. Figure 2d shows the reverse pole diagram corresponding to A1000 HEA. The texture caused by cold rolling was not completely eliminated, and the grain orientation tended towards to the <111> grain plane. Praveen [26] studied the recrystallization behavior of CoCrFeNiMn HEA and found that this HEA can form equiaxed grains after annealing at 700 °C. In this research, due to the formation of σ phases, the activation energy of the grain growth in HEA was higher than CoCrFeNiMn HEA, which inhibited the growth of the FCC phase and caused the temperature of complete recrystallization to increase to 1000 °C.

**Figure 3:**
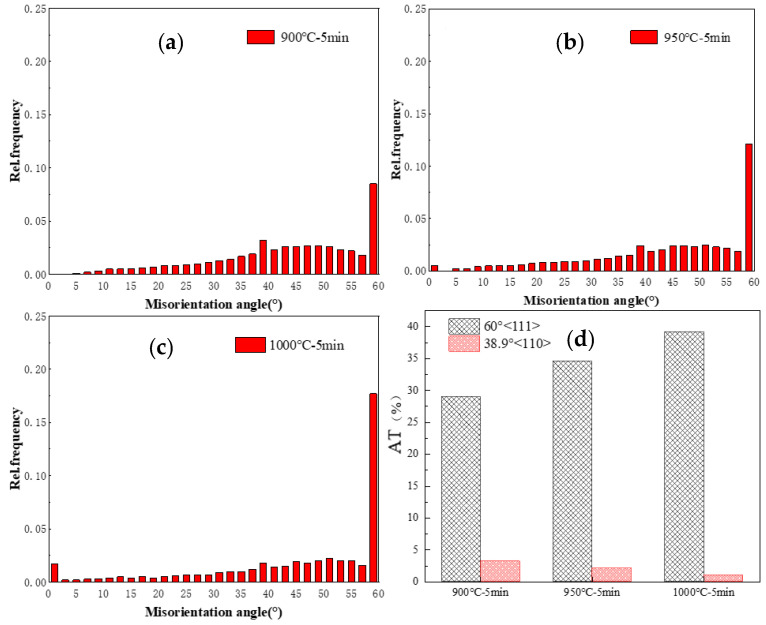
Misorientation angle distribution maps. (**a**) A900, (**b**) A950, (**c**) A1000. (**d**) Volume fraction of AT with different orientations.

Figure 3 shows the misorientation angle distribution maps of HEA. The densities of the subgrain boundary dislocations formed after cold rolling were relatively higher. Migration occurred after annealing, and it transformed into a large angle grain boundary. Therefore, the main characteristic of the HEA is a large number of large angle grain boundaries corresponding to 15° to 58° RD. Additionally, the peaks corresponding to the 0–2° substructure also slightly increased as the annealing temperature increased, indicating the presence of a small amount of substructure in A1000 HEA. It was also observed that the peaks of 38.9° and 60° were significantly higher. The 60° peak is generally defined as a 60°<111> orientation Σ3 first-order twin boundary, and the 38.9° peak is defined as a 38.9°<110> orientation Σ9 s order twin boundary. According to Otto et al. [27], the volume fraction of Σ3 twin boundaries increases when the annealing temperature increases, while the volume fraction of Σ9 twin grain boundaries decreases with the increase in the annealing temperature. As can be seen from Figure 3d, the increase in the volume fraction of the annealing twins was directly proportional to the grain size of the recrystallized grains. Accordingly, the 60°<111> orientation annealing twins in A1000 HEA had the highest volume fraction.

**Figure 4:**
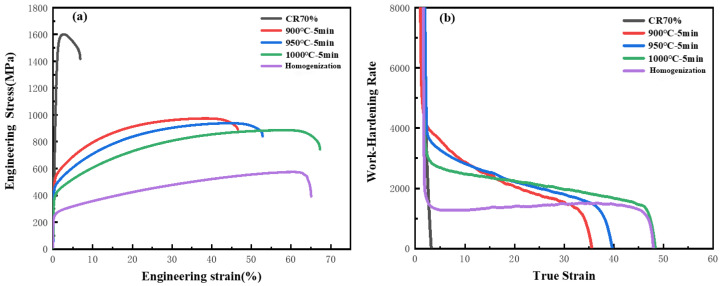
The room-temperature tensile curve of the annealed HEA. (**a**) Engineering stress–strain curve, (**b**) work hardening rate curve.

Figure 4a shows the room-temperature tensile curve of the annealed HEA. It can be observed that the tensile properties significantly improved compared to homogenization HEAs. The tensile strength of the CR70 HEA was 1600 MPa, but the elongation was only 6%. Due to the uneven and smaller average grain size, the yield strength and tensile strength of the A900 HEA increased significantly to 458 MPa and 938 MPa, respectively. After annealing at 1000 °C, the tensile strength decreased to 885 MPa, and the elongation increased to 68%, higher than that of homogenized HEA. According to Figure 4b, the work hardening rate of the annealed HEAs gradually decreased with the increase in the true strain. However, the work hardening rate of A1000 HEA showed a significant increase compared to other annealed HEAs after 20% true strain. The grain refinement in A900 and A950 limited the formation of annealed and deformation twins, while the increased annealed and deformation twins in A1000 HEA improved the work hardening rate.

**Figure 5:**
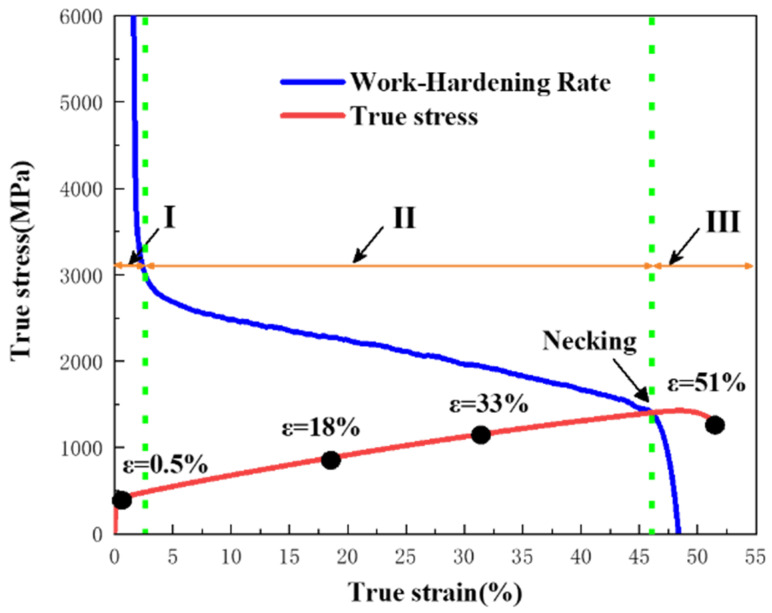
True stress–strain curve and work hardening rate curve of A1000 HEA.

**Figure 6:**
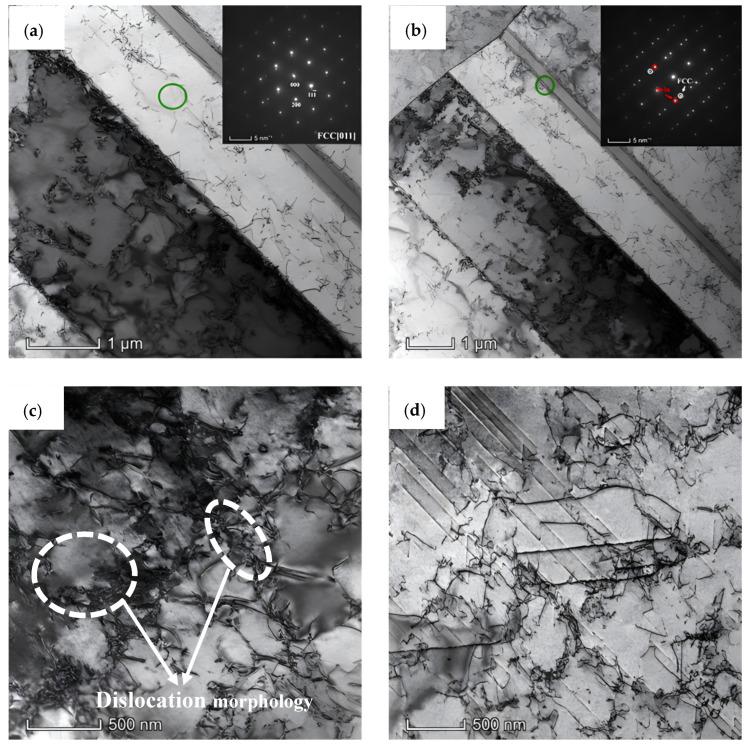
TEM images of A1000 HEA. (**a**,**b**) Bright field image (the insets are selected diffraction patterns of the green circle); (**c**,**d**) dislocation morphology.

**Figure 7:**
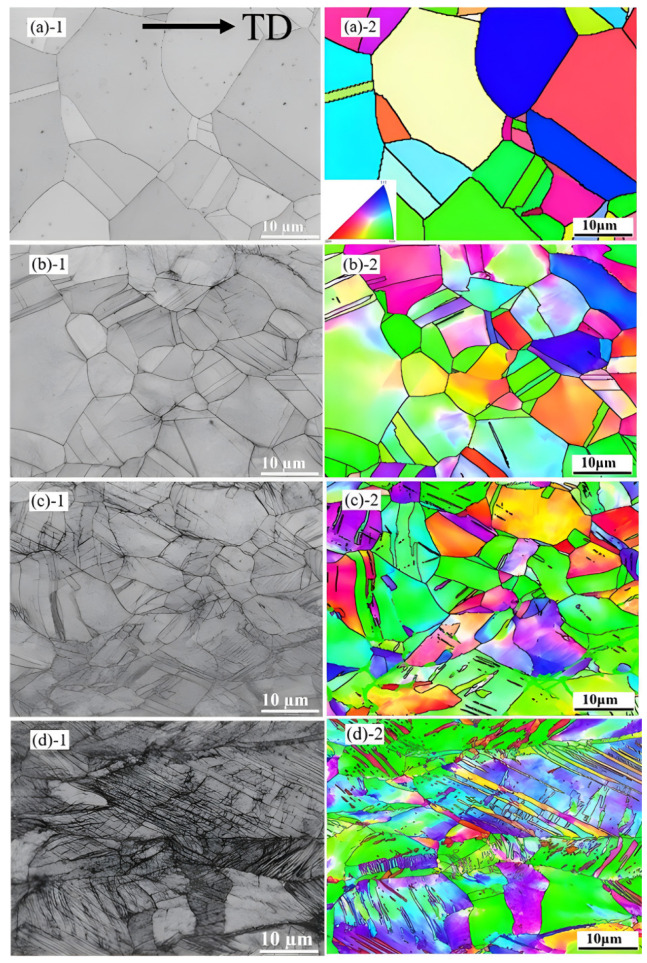
EBSD-BC diagrams of the A1000 HEA under different strains. (**a-1**,**a-2**) ε = 0.5%, (**b-1**,**b-2**) ε = 18%, (**c-1**,**c-2**) ε = 33%, (**d-1**,**d-2**) ε = 51%.

In order to study the deformation mechanism of A1000 HEA, the true stress–strain curve was divided into three stages based on the inflection point of the work hardening rate in Figure 5. Figure 6 shows the TEM images of A1000 HEA before tensile deformation. The A1000 HEA reached complete recrystallization and formed equiaxed grains with different orientations. At the same time, the A1000 HEA eliminated the strong texture caused by cold deformation and presented a random texture. The illustrations in Figure 6a,b prove that the strip structure was the annealing twin, and the dislocation pile-up was generated at the grain boundaries of annealing twins. It can be seen from Figure 6c,d that the A1000 HEA contained a large number of dislocation cells and dislocation entanglements. Figure 7 show the EBSD-BC diagram and corresponding IPF diagram of the A1000 HEA under different true strains. At 0.5% true strain, deformation twins did not formed, and the plastic deformation was mainly dominated by dislocation slipping [28]. With the further increase in strain, the grains were severely stretched in the direction of the tensile axis, and the relative content of the small angle grain boundary increased gradually. The deformation mechanism was controlled simultaneously by the dislocation slip and deformation twins. When ε = 51%, deformation twins occupied the positions of annealing twins and inhibited the dislocation movement. It can be seen from the IPF diagram that a large lattice rotation occurred during the tensile deformation process. Deformation twins were mainly formed at grain boundaries of <111> and <100> grain orientations, and mainly expanded along the <111>//TA orientation (//TA is defined as parallel to the direction of the tension axis).

## 4. Conclusions

The as-casted (Fe50Mn30Co10Cr10)97C2Mo1 HEA was annealed at different temperatures after CR70%. As the annealing temperature increased, the phase composition remained a single FCC structure. The A900 and A950 HEA exhibited uneven grain sizes and rich σ precipitation phase at grain boundaries. The grains began to grow and complete recrystallization, and no σ phases were observed in A1000 HEA. The elongation (EL) of A1000 HEA was up to ~68%, and the tensile strength (UTS) was ~885 MPa. There were higher volume fractions of the 60°<111> orientation annealing twins in A1000 HEA, and the <001> + <111> double texture formed with the increase in deformation. Accordingly, the TWIP effect, as the main deformation mechanism, finally replaced the dislocation and twin mechanism.

## Data Availability

Data will be made available upon request.
